# Understanding patient experiences of a community-based intervention to improve bowel screening uptake: a mixed-method evaluation of Call for a Kit clinics

**DOI:** 10.1136/bmjopen-2025-109731

**Published:** 2026-03-24

**Authors:** Shahida Hanif, Rebecca Jefferson, Robert Kerrison, Sandro T Stoffel, Stephen Rowley, Lorraine Morris, Christian von Wagner, Yasemin Hirst

**Affiliations:** 1 Blackpool Teaching Hospitals NHS Foundation Trust, Blackpool, UK; 2 Applied Health Research Hub, University of Lancashire, Preston, UK; 3 School of Health Sciences, University of Surrey, Guildford, UK; 4 Institute for Pharmaceutical Medicine, University of Basel, Basel, Switzerland; 5 Research Department of Behavioural Science and Health, University College London, London, UK; 6 Patient Representative, Public Co-Applicant, UK

**Keywords:** Early Detection of Cancer, Health Equity, Patient Navigation, Patient Satisfaction, Surveys and Questionnaires, QUALITATIVE RESEARCH

## Abstract

**Abstract:**

**Objectives:**

The study explored patient experiences of the Call for a Kit (CFAK) intervention, a community-based initiative designed to improve bowel cancer screening uptake and examined the mechanisms that may support participation among non-responders.

**Design:**

A convergent parallel mixed-methods design was employed, combining quantitative surveys and qualitative interviews.

**Setting:**

The evaluation was conducted in general practices across Lancashire and South Cumbria, Northwest England, where CFAK clinics were delivered by an external health promotion team based within the Community Voluntary Services. These clinics target practices with low screening uptake.

**Participants:**

A total of 113 CFAK attendees aged 54 and above, and who had missed their most recent screening invitation, completed a patient experience survey. 12 participants were purposively sampled for follow-up interviews.

**Outcome measures:**

Statistical analyses examined associations between patient experience and screening behaviours, including kit ordering and intention to complete the screening kit. Thematic analysis explored barriers and facilitators to participation, as well as experiences of CFAK clinics.

**Results:**

Patient experience scores were significantly higher among women than men and were positively associated with intention to complete the kit, though not with kit ordering. Qualitative findings indicated that CFAK addressed key barriers such as low awareness, confusion and emotional discomfort by providing personalised education, reassurance and culturally sensitive support. Participants particularly valued the relational aspects of the intervention, including the face-to-face delivery and communication in preferred languages.

**Conclusions:**

CFAK clinics appear to enhance psychological capability and motivation for bowel screening by offering tailored, inclusive and supportive care. These findings highlight the value of patient-centred approaches in addressing inequalities in cancer screening and offer insights for the design of future community-based interventions.

**STRENGTHS AND LIMITATIONS OF THIS STUDY**

- This study employed a mixed-methods design, combining quantitative and qualitative data to provide a comprehensive understanding of patient experiences.
- The evaluation was embedded within routine service delivery, enhancing ecological validity and relevance to real-world practice.
- Recruitment was conducted across multiple general practitioner practices in Lancashire and South Cumbria, enhancing contextual relevance but limiting geographic diversity.
- Data collection tools were co-developed with stakeholders to ensure relevance and clarity.
- Limitations include potential selection bias due to voluntary participation and limited generalisability beyond the study population.

## Introduction

 Colorectal cancer (CRC), also known as bowel cancer, is the fourth most common cancer in the UK and was the second leading cause of cancer-related death between 2017 and 2019.^1^ Although many CRC cases are preventable through early detection, diagnoses often occur at a symptomatic stage, when the disease is more advanced and treatment is less effective. In the UK, the National Bowel Cancer Screening Programme (BCSP) aims to detect CRC early, before symptoms develop, by offering all adults aged 50–74 a home-based stool sampling test, the faecal immunochemical test (FIT). The FIT was introduced in 2019 as a more user-friendly test than its predecessor: the faecal occult blood test (FOBt).

Since the introduction of the FIT, screening uptake in England has improved to 70.2% (2022–23).^2^ However, regional disparities persist. Uptake in Northwest England remains slightly lower (68.2%), with pronounced variation between the most deprived areas (57%) and the least deprived areas (77.1%).^3^ At the local level, uptake varies widely between Primary Care Networks and general practitioner (GP) practices, with some reporting levels as low as 35%.^4^ These inequalities are influenced by sociodemographic factors, with lower participation observed among ethnically diverse communities, individuals living in deprived areas and vulnerable groups.^5 6^

To address these disparities, NHS England Northwest commissioned a community engagement initiative in 2015 called ‘Call for a Kit’ (CFAK). CFAK targets GP practices with screening uptake below 60%, contacting non-responders by phone and offering them a consultation, either in person or remotely, to discuss barriers to screening participation and provide the opportunity to order a replacement kit. Quantitative evaluations have shown CFAK to be effective, with over 14% of targeted non-responders requesting, completing and returning a kit.^7^

Despite evidence of CFAK’s impact on uptake, its effectiveness from the patient perspective has not yet been explored. Evaluating patient experience is essential for understanding how interventions are received, particularly in underserved populations where trust, cultural relevance and communication barriers may influence engagement.^8^ Insights into patient perspectives can inform improvements to service delivery and support the equitable implementation of screening programmes. Understanding how patients experience CFAK may offer insights into why the intervention works for some individuals and not others and inform improvements or adaptations for other cancer screening programmes.

This study aimed to explore patient experience of CFAK using a convergent parallel mixed method design with a quantitative and qualitative part chosen to capture both the measurable outcomes of patient experience and the nuanced, contextual factors that influence screening behaviours. This design enables a more comprehensive understanding of how CFAK operates and for whom it is most effective through collecting different but complementary data relating to patient experience.^9^

### Research questions

The research was guided by the following research questions:

1. Quantitative component: Is patient experience of CFAK associated with ordering and intention to complete a kit?
2. Quantitative component: Are there differences in patient experience scores by sociodemographic characteristics?
3. Qualitative component: How does CFAK address barriers to participation in bowel cancer screening?
4. Mixed-methods comparison: How do interviews with CFAK attendees explain the associations between patient experience and ordering and intent to complete a kit?
5. Mixed-method comparison: How do interviews with CFAK attendees explain differences in patient experience by sociodemographic characteristics?

## Methods

### Design

A convergent parallel mixed-methods evaluation was conducted with CFAK clinic attendees between January and May 2024 to explore patient experience of the intervention. A pragmatic epistemology^10^ was employed to gain a better understanding of CFAK delivery to evaluate patient experience outcomes using quantitative and qualitative data collection methods. Quantitative data were collected via patient experience surveys and qualitative data were gathered through semistructured interviews. For both parts, data were collected during the same time period, analysed independently, then results triangulated to provide a comprehensive understanding of how CFAK supports bowel screening uptake from patient perspectives. The manuscript was drafted using the Strengthening the Reporting of Observational Studies in Epidemiology (STROBE),^11^ COnsolidated criteria for REporting Qualitative research (COREQ)^12^ and Good Reporting of A Mixed Methods Study (GRAMMS)^13^ reporting guidelines and checklists, to ensure thorough reporting of the quantitative and qualitative elements and the overall mixed-method design (online supplemental tables 1–3).

### Setting

The evaluation was conducted across GP practices in Lancashire and South Cumbria, Northwest England, identified as having CRC screening uptake rate below 60%. These practices were part of the regional implementation of the CFAK intervention, commissioned by NHS England Northwest to improve screening participation among non-responders. In England, the CRC screening episode automatically closes after 13 weeks if the individual does not return their screening kit. Their GP practice is informed of the non-participation, and a non-responder read code is added on the patient’s electronic health record. When health promotion officers (HPOs) start working with a low uptake GP practice, the practice runs a search for the non-responder read code and HPOs use this list to check patient eligibility for CRC screening and invite eligible patients to a CFAK clinic via telephone. CFAK clinics were delivered either in person at GP surgeries or remotely via telephone, depending on patient preference and availability. All data collection activities (surveys and interviews) were embedded within routine CFAK service delivery, ensuring ecological validity and relevance to real-world practice.

### Participants

#### Survey participants

All individuals who attended a CFAK clinic between January and June 2024 were eligible to participate in the patient experience survey. Inclusion criteria were: aged 54 or older, registered with a GP practice in Lancashire or South Cumbria with screening uptake below 60% screening, and not completed their most recent screening invitation (ie, screening non-responder). The age limit of 54 was used to align with the eligibility criteria for CRC screening in Lancashire & South Cumbria at the time of the study due to the staged roll-out of the age extension in the BCSP.^14^ Exclusion criteria included individuals currently under surveillance, diagnosed with CRC or excluded from the national screening programme. These criteria also make individuals eligible to be invited to a CFAK clinic, and thus all individuals who attended a CFAK clinic were eligible to take part in the patient experience study. Participants were invited to complete the survey at the end of their CFAK consultation (in-person or telephone). Surveys were available in English, Urdu and Gujarati and were distributed via paper, text message or post. No incentives were offered.

#### Sample size estimations

No formal size calculations were performed to establish statistical power as this was an exploratory study. However, we estimated the potential target population based on the number of clinics being carried out on a weekly basis. Approximately 10 non-responders are seen in each face-to-face clinic, four clinics are delivered each week by four CFAKC officers (40 consultations per week × 4-week average per month × 6 months=960). As a result, we estimated approximately 1000 individuals would be targeted to participate in the survey. Based on past BCSP Patient Experience Questionnaire (PEQ) response rates,^15^ we estimated 60%–70% of the questionnaires to be returned.

### Interview participants

Qualitative participants were recruited from the survey sample opportunistically. At the end of the survey, an Expression of Interest (EoI) form invited respondents to indicate willingness to participate in a follow-up interview. Those who returned the EoI were contacted by the research team. We received 28 EoIs for willingness to have interviews. A few contact details were not readable to the researcher, or the numbers were not working. Two phone call attempts were made to the remaining EoIs. A total of 12 participants responded, provided written consent and took part in one-to-one interviews conducted via phone or Microsoft Teams. Participants received a £20 shopping voucher as a thank you for their time. Sample size was determined when data saturation was reached.

### Data collection

#### Survey data

Quantitative data were collected using a co-developed questionnaire, designed in collaboration with CFAK team members to capture demographic characteristics, previous screening history, barriers to participation and patient experience of the CFAK clinic. The questionnaire included 14 tailored barrier items, informed by the Capability, Opportunity and Motivation Behaviour change (COM-B) model^16^ and local service knowledge. Patient experience was assessed using seven statements on a 5-point Likert scale (‘strongly disagree’ to ‘strongly agree’) covering (1) overall satisfaction, (2) having enough time with the officer, (3) having questions answered, (4) feeling better informed about BCSP, (5) having their values, culture and religion heard, (6) personalised advice and support and (7) having concerns addressed. These items were derived from the existing BCSP patient-reported outcome measures questionnaires which were at the time being co-developed and piloted in collaboration with the BCSP.^17^ These were discussed with the CFAK team members to select and adapt into context and feedback was provided by patient representatives. For instance, we adapted “After speaking with the Specialist Screening Practitioner, I felt better informed about the potential outcomes of my colonoscopy’,” as “I felt better informed about the bowel screening programme”.

Demographic information collected included age in categories, gender, existing mental health conditions, existing long-term conditions and belonging to inequality groups (see table 1 for response options). Inequality groups were identified based on local-level inequalities associated with BCSP participation and the CORE20Plus5 criteria,^18^ and participants were able to select all options that applied to them. This question was intentionally designed to minimise participant burden, avoid binary ethnic classifications (eg, white/non-white ethnicities) in small samples, and capture intersectionality more efficiently than traditional sociodemographic measures (eg, 2021 Census includes 19 ethnicities in 5 categories).^19^

**Table 1:** Description of the study sample (N=113)

	N	(%)
Gender Which of the following options best describes you?		
Women	55	(48.7)
Men	48	(42.5)
Non-binary	2	(1.8)
Prefer not to say/missing	8	(7.1)
Age Please select your age category		
45–54	2	(1.8)
55–64	62	(54.9)
65–74	38	(33.6)
Prefer not to say/missing	11	(9.7)
Existing mental health condition Do you have an existing mental health condition?		
Yes	17	(15.0)
No	81	(71.7)
Prefer not to say/missing	15	(17.3)
Long-term health condition Do you have any long-term conditions?		
Yes	48	(42.5)
No	47	(41.6)
Prefer not to say/missing	18	(15.9)
Inequalities Please can you state if you describe yourself as part of one of the following groups (select all that apply)		
Particular ethnic minority groups (Asian/Asian British)	24	(21.2)
Particular ethnic minority groups (Black/African/Caribbean/Black British)	4	(3.5)
People with disabilities	6	(5.3)
Carers	9	(8.0)
Lesbian, gay, bisexual, transgender, queer people	1	(0.9)
Refugee/Asylum seeker	0	–
People who are homeless	0	–
People with language barriers	1	(0.9)
Blind or visually impaired	0	–
d/deaf or hearing impaired	6	(5.3)
Living in a multigenerational household	1	(0.9)
None of the above	48	(42.5)
Prefer not to say/missing	13	(11.5)

The survey included general questions around CFAK attendance, including whether they attended a face-to-face or telephone clinic, how happy they were to be invited and how easy it was to understand the invitation rated using 5-point Likert scales (‘not at all’ to ‘very much’) and the reasons for attending the clinic selected from six options. Participants were also asked whether they ordered a test afterward (‘yes’, ’no’ or ’already have one at home’) and rated how likely they were to complete the new kit (5-point Likert scale: ‘very likely’ to ‘not at all likely’).

At the end of the PEQ, an EoI form was included for respondents interested to take part in one-to-one interviews. Completed surveys were returned to the CFAK team based at Blackpool Victoria Hospital NHS Trust using prepaid envelopes. All surveys were anonymised prior to data entry into a secure NHS survey platform.

#### Interview data

Qualitative data were collected through semistructured interviews with a subset of survey respondents who expressed interest via the EoI form. Participants were contacted by the research team and interviews were scheduled based on availability and preference. The interviewer was a BCSP Health Promotion Specialist (SH) who identifies as a woman, received interview training prior and had no prior relationships with the participants. Interviews were conducted via telephone or Microsoft Teams and audio-recorded with consent.

The interview guide was developed to further explore topics measured on the survey including participants’ experiences with the BCSP prior to CFAK, their perceptions of the CFAK invitation and consultation, and any subsequent screening behaviours (online supplemental material S1). Feedback was received from the patient representative on the clarity and usefulness of the interview questions. Interviews lasted approximately 30–45 min, and participants received a £20 shopping voucher as a thank-you. Only the participants and interviewer were present during the interviews. Data saturation was determined during data collection through discussions between SH and YH, where it was determined that interviews were not eliciting any new information related to the research questions.^20 21^

### Data analysis

#### Quantitative analysis

Quantitative data were analysed using SPSS V.29.0. Descriptive statistics summarised participant characteristics, previous screening history, reported barriers to participation, CFAK clinic type and behavioural outcomes (kit ordering and intention to complete). Responses to the seven patient experience items were aggregated to generate a composite score (range: 7–35), with higher scores indicating a more positive experience. Only participants with complete responses to all seven items were included in the total score calculation.

Exploratory factor analysis was conducted to examine scale structure, and internal consistency was assessed using Cronbach’s alpha. One-way analyses of variance (ANOVAs) and Welch’s ANOVA (where assumptions of normality, ie, Levene’s test, were violated) were used to examine differences in patient experience scores across demographic and health-related characteristics. Logistic regression analyses assessed whether patient experience scores predicted two binary outcomes (1) ordering a test kit and (2) intention to complete the kit. The latter was derived from Likert-scale responses to the item ‘Likelihood to complete kit’ (1=‘not at all’ to 5=‘very likely’), with ‘very likely’ coded as ‘high intent’ and all other responses as low intent. Analyses were conducted using complete case analysis, statistical significance was set at p*<*0.05 and for effect sizes omega squared (ω)^2^ was used for ANOVAs^22^ and OR for logistic regression.

#### Qualitative analysis

Following completion of all interviews, audio recordings were transcribed verbatim. Braun and Clarke’s^23^ thematic analysis, where a thematic representation of patterns and nuances in the dataset is constructed. Given the exploratory nature and emphasis on patient perspectives, an inductive analysis approach was taken rather than a theoretically driven approach. Three authors (SH, YH and RJ) independently reviewed transcripts to familiarise themselves with the data. Transcripts were reviewed line-by-line, and inductive codes were developed to summarise key content. Coding was conducted by RJ using NVivo V.14.0. Related codes were grouped into preliminary themes, which were refined collaboratively through discussion among SH, YH and RJ and re-examination of associated data. Consistent with the reflexive nature of thematic analysis,^24^ themes were developed and refined collaboratively based on shared reflexive interpretations, rather than using a narrower approach to establish ‘coder reliability’. Final themes were reviewed and refined by all authors to ensure consistency, relevance, and alignment with the research objectives.

#### Reflexivity

Each researcher involved in the qualitative analysis had different experiences and expertise. RJ is an early career postdoctoral researcher with experience of both qualitative and quantitative evaluations and, at the time of this research, was new to the area of cancer and cancer screening. SH is a Health Promotion Specialist for the BCSP in England and is a part of the CFAK team. YH is a Reader in Behavioural Sciences and Health, bringing expertise in cancer screening and behaviour change. RJ led the thematic analysis without any bias from previous understandings of the cancer screening literature or CFAK to ensure YH and SH’s values and professional experience did not influence the interpretation of the data. Initial themes were discussed with SH and YH for their expertise to ensure nuance was not lost.

#### Mixed-methods analysis

After the independent analysis of the quantitative and qualitative data, the results were triangulated by integrating related findings by the study’s mixed-method research questions. Joint display was used to display findings and interpretations side-by-side.^25^ Integration was led by RJ and reviewed by YH, SH and SS.

### Patient and public involvement

Patient and public involvement was embedded throughout the study. A patient representative contributed to the design and refinement of the PEQ, ensuring that items were relevant, accessible and reflective of patient priorities. Additionally, questions from the PEQ were discussed with members of a local community group, who provided feedback and shared their perspectives. SR contributed to the interpretation of findings and reviewed the final manuscript. These contributions informed the development of the evaluation tools and interpretation of findings. While patients were not directly involved in recruitment or data collection, their views were incorporated through these engagements and through qualitative interviews with CFAK attendees.

## Results

### Quantitative survey findings

Of the 500 surveys distributed, 113 were returned (response rate: 23%). As shown in table 1, 48.7% of participants identified as women, 54.9% were aged 55–64, 71.7% reported no mental health condition, 42.5% had a long-term condition, and 42.5% did not identify with any specified inequality group.

Regarding previous participation in the BCSP, 46.9% (n=53) reported never completing a kit, 20.0% (n=22) had completed some previously, and 17.0% (n=19) had completed all except their most recent kit. Table 2 presents the barriers to CRC screening endorsed by participants. Most respondents (76.1%, n=86) reported at least one barrier, with the most common being not knowing how to complete the kit (22.1%; n=25) and forgetting to do so (20.4%; n=23).

**Table 2:** Barriers endorsed by participants

Barriers to CRC screening participation*	N endorsed (%)
I did not know how to complete kit	25 (22.1)
I forgot about it	23 (20.4)
Not received the kit	13 (11.5)
I did not have symptoms of bowel cancer	12 (10.6)
I lost the kit	11 (9.7)
I had more important things to worry about	10 (8.8)
I did not feel at risk of bowel cancer	9 (8.0)
I found completing the test kit too messy	7 (6.2)
I would rather not want to know if I have bowel cancer	6 (5.3)
The test kit is too fiddly for me	4 (3.5)
I was too worried about what other tests I might have	4 (3.5)
I did not have time to complete the kit	2 (1.8)
If someone is meant to get bowel cancer, they will get it no matter what they do	1 (0.9)
If someone has bowel cancer, it is already too late to get treated	0
It is at odds with my religion and culture	0

* Question wording was ‘Our records indicated that you have not returned the test kit the last time you received it. We would be very grateful if you could tick all that apply to your reason why you did not.

CRC, colorectal cancer.

**Figure 1:**
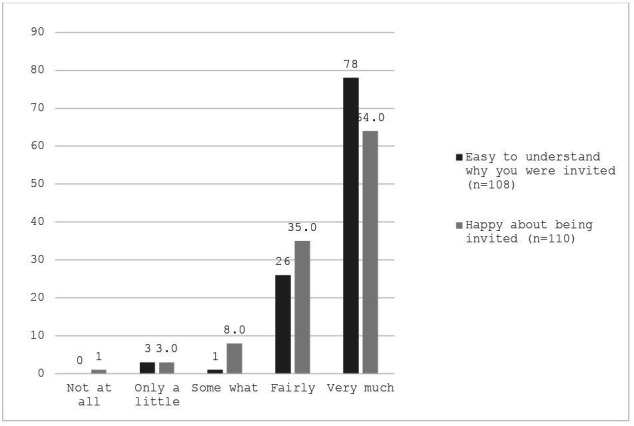
Bar chart showing extent of ease of understanding and happiness of being invited to CFAK. CFAK, Call for a Kit.

Most participants (97%; n=110) attended an in-person CFAK consultation, while 1.8% (n=2) received a phone consultation. As shown in figure 1, the majority reported understanding why they were invited (fairly=23.0%; very much=69.0%) and were happy to be invited (Fairly=31.0%; Very much=56.6%). The most common reasons for attending included being invited by their GP surgery (45.1%), wanting to know more about the kit (27.4%) and learning about the BCSP (27.4%). Other reasons included needing a replacement kit (15.9%), believing attendance was compulsory (12.4%) and experiencing symptoms they wished to discuss (4.4%).

Exploratory factor analysis of the seven patient experience items revealed a single factor accounting for 72.7% of the variance (online supplemental material S2). Internal consistency was excellent (Cronbach’s α=0.92), supporting the use of a composite score. Participants with missing experience scores (n=20) were excluded from subsequent analyses.

The overall mean patient experience score was high (M=32.29, SD=3.34, range: 23.00–35.00). Welch’s ANOVA revealed a significant difference by gender, with women reporting a more positive experience (M=33.40, SD=2.52) than men (M=31.23, SD=3.74), F(1,64.25)=9.55, p=0.003, ω^2^=.097 (95% CI 0.004 to 0.228). Due to the small number of non-binary participants, only women and men were included in this comparison. No significant differences were observed across age categories, inequality groups, or between participants with and without a mental health condition or a long-term condition. See table 3 for detailed comparisons.

**Table 3:** Mean patient experience score by participant characteristics and ANOVA significance level

Participant characteristics	N	Mean (SD)	P value*	ω^2^ (95% CI)
Gender			0.003*†	0.097 (0.004 to 0.228)
Woman	48	33.40 (2.52)		
Man	39	31.23 (3.74)		
Age			0.374†	0.004 (−0.012 to 0.070)
55–64	57	32.30 (3.46)		
65–74	30	32.90 (2.70)		
Inequality group			0.125	0.034 (−0.037 to 0.135)
None	39	32.31 (2.90)		
BAME	24	33.17 (2.91)		
Disability	12	30.58 (4.17)		
Carers	9	33.11 (3.26)		
Mental health condition			0.617†	0.009 (−0.013 to 0.056)
No	65	32.63 (2.99)		
Yes	16	33.06 (3.07)		
Long-term condition			0.053†	0.035 (−0.013 to 0.153)
No	40	33.13 (2.81)		
Yes	39	31.67 (3.70)		

* P value is significant based on 0.05.

† Welch’s ANOVA p value reported.

ANOVA, analysis of variance; BAME, Black, Asian, and Minority Ethnic.

Most participants (79%, n=89) ordered a new kit, and 91% (n=103) reported being likely to return it. Logistic regression showed no significant association between patient experience scores and kit ordering (OR=1.07, 95% CI 0.92 to 1.26, p=0.383). However, higher experience scores were significantly associated with high intent to complete the kit (OR 1.88, 95% CI 1.28 to 2.77, p=0.001). Participants with high intent scored higher (M=33.00, SD=2.84) than those with low intent (M=27.60, SD=2.07).

### Qualitative interview findings

Of the 113 participants who returned a survey, 12 also took part in a follow-up interview. Their mean age was 64.2 years (range: 59–70), with equal representation of men and women (n=6 each). Most participants identified as White (n=10). Thematic analysis generated three overarching themes:

1. Barriers to bowel screening prior to CFAK.
2. How CFAK helped address these barriers.
3. Relational and contextual factors that made CFAK effective.

### Barriers to bowel screening

Participants described a range of barriers that had previously prevented them from engaging with CRC screening. A common issue was low awareness and understanding of the screening programme and the FIT kit. Some had never heard of the BCSP:

Before the clinic, I had never heard about the BCSP. (P09)

Others indicated that they had never seen a kit before or mistook the kit for something unrelated such as a COVID-19 test:

I don’t remember really just looked like a Covid box. (P01)

Uncertainty and confusion about how to complete the kit was also a common deterrent:

People do not do the kit as they get scared - like how to do it, what to do etc. (P09)

Several participants reported discarding correspondence from the BCSP, mistaking it for junk mail or being unsure of its purpose:

I didn’t know what to do with it, so I just binned it. (P08)

A lot of letters you get are circulars. I don’t look at them, they go in the bin. (P10)

Negative past experiences with the older FOBt kit and lack of awareness about the updated version also contributed to avoidance:

I never opened the kits… I didn’t like the first one, so I didn’t trust it. (P12)

Other barriers included lack of time and low perceived personal risk of having CRC:

I think some of them don't do the kits because they don’t have time…and they think it will not happen to them. (P03)

Negative feelings and concerns towards completing the screening kit and of the consequences of this were evident from participants’ responses. Some participants expressed discomfort with the nature of the test and a fear of a positive result:

Sometimes you think ‘God’, you know, cause it’s just not a nice area and you don't know what to do. And it’s easy to just ignore it and think that ‘I'm alright I won’t have it done’. (P02)

I was scared… that if I did it and it came back saying that it was something then I don’t think I would’ve coped with that…with everything else that’s been going on over the last couple of years*.* (P07)

### How CFAK addresses barriers

Participants described how CFAK helped overcome these barriers to screening by improving their understanding of bowel cancer and the screening process. Education provided during consultations clarified the purpose of the FIT kit and why it was sent, which helped alleviate confusion and hesitation:

I didn’t do the kit as I didn’t know what it was and what it was for… What she said helped me… made complete sense and I thought why didn’t I do it earlier? (P06)

This increased understanding led some participants to becoming advocates for screening:

They are wonderful…its getting across to people how important it is to have a bowel screening test…I told people if they get a bowel screening call or letter to go to it…which they all said they would. (P01)

The clinics also addressed fears about receiving a positive result by providing reassurance and information about early detection and treatment:

It’s brilliant, it’s given me peace of mind… It’s helped me so much-it’s one of those cancers that if caught early it can be treated. (P04)

Clear instructions on how to complete the kit helped dispel concerns about messiness and complexity, especially in comparison to the older FOBt:

It’s so easy to do the test whereas my perception of the old test was messy and hard to do…I tell everyone that they’ve got to it, its brilliant. Before I would not tell anyone-now I will. It’s changed my whole perception*.* (P12)

However, for one participant, the support provided was not enough to overcome personal challenges and emotional barriers:

…I’m just not in the right frame of mind. Like I said, if [the kit] came back, and it said anything was wrong I don’t think I would cope very well with it. (P07)

### Relational and contextual factors that make CFAK effective

Participants described how the CFAK clinics provided a positive and supportive healthcare experience, shaped by both the structure of the service and the qualities of the HPO delivering it. The service was widely praised, with some participants sharing their positive experience with others:

I was telling my friend how good it was, my God, you know. I honestly and genuinely thought it was a good service. (P02)

The HPOs were described as friendly, professional and knowledgeable, which helped participants feel comfortable and informed:

I felt welcomed, she was friendly, and she was informative, and she explained everything… she was really good. (P04)

She was very friendly, she made me feel at ease and whatever she said seemed to just sink in…. And I didn't feel any anxious. (P03)

Participants appreciated the non-pressurising and informal approach:

it was informal, he didn’t pressurise me… put me at ease straight away and yeah, a good experience. (P01)

CFAK was also seen as responsive to individuals’ needs, offering personalised care. Some participants initially felt anxious when contacted by their GP practice, but this was quickly alleviated by the HPO’s reassurance:

when we get a phone call from the doctors or NHS….we just panic… The [officer] that rung from the doctors put my mind at rest…she explained what it was, and we said ok yes no problem. (P06)

There were mixed preferences regarding how participants were invited to clinic. While one participant preferred a letter, most favoured a phone call for its personal touch and immediacy:

I think a letter [is the best way to invite people to the clinic]. (P01)

it’s easy to just ignore it… but because it was that interaction that contact to say, ’well, you come into our office’… I had time to do that. (P02)

Face-to-face interaction was especially valued for enabling real-time clarification and support:

If I’d had a leaflet, I would not have done the kit…I could talk to that lady, and she answered my questions. (P11)

Participants also noted that the clinics created a safe space to ask questions they might otherwise avoid:

…any questions I had to ask were answered. It’s questions that I think some people want to ask but they don’t, or they don’t know how to ask them. (P05)

Information was delivered in an accessible way, tailored to the individual’s level of understanding:

She was so clear… she explained it to me in lay man terms and it didn’t sound medical…so it works. (P12)

Finally, the ability to conduct the clinic in participants’ preferred language was seen as a crucial factor in accessibility:

This is a very good way of talking and showing people the kit especially talking in their own language. This so important. (P09)

### Triangulation of quantitative and qualitative findings

Table 4 shows the triangulation and interpretation of the quantitative and qualitative findings using joint display.

**Table 4:** Joint display of triangulation of quantitative and qualitative analysis by mixed-method research question

Mixed-method research questions	Quantitative finding	Qualitative finding	Merged finding
What barriers do non-responders face when deciding to participate in CRC screening?	Most common barriers endorsed by participants were: Not knowing how to complete the kit (22.1%) Forgetting to complete the kit (20.4%) Not receiving the kit (11.5%) Having no symptoms (10.6%) Lost the kit (9.7%) Had more important things to worry about (8.8%) Do not feel at risk of bowel cancer (8.0%) Found completing the kit messy (6.2%)	Theme 1: Barriers to bowel screening prior to CFAK Participants explained that they had not completed their previous kit due to the following barriers CRC screening: Low awareness and understanding of the screening programme and FIT, including not knowing how to complete the kit Mistaking kit for COVID test and correspondence as junk mail Lack of time and personal risk Discomfort with the nature of the test Fear of receiving a positive result and the subsequent tests needed	The barriers identified in the quantitative survey and qualitative interviews converge. The interview findings provide a more in-depth understanding of the barriers to CRC screening and provide explanation as to how some barriers occur. For example, in the surveys, 9.7% of respondents indicated that they did not complete their previous screening due to losing the kit, and 11.5% reported not receiving the kit. In the interviews, participants explained that the correspondence from the BCSP had been mistaken as junk mail and so thrown away. This, coupled with a lack of awareness of the BCSP, suggests that some people are throwing away the screening kit because they are not aware that they are receiving a screening kit and why they are receiving it.
How do interviews with CFAK attendees explain the associations between patient experience and ordering and intent to complete a kit?	Patient experience scores were significantly associated with intention to complete the screening kit (OR 1.88, 95% CI 1.28 to 2.77, p=0.001), though not with kit ordering (OR=1.07, 95% CI 0.92 to 1.26, p=0.383).	Theme 2: How CFAK helped address barriers to bowel screening Participant’s accounts indicated that CFAK clinic provides non-responders with the knowledge, skills, support and confidence to complete the kit but for one participant this was not enough to overcome their barrier of fear of receiving a positive test and any subsequent tests that would be needed, due to their other unrelated personal concerns.	The quantitative finding indicates that a positive experience encourages attendees to want to complete the kit. The qualitative explains this association by showing that CFAK makes people intend to complete the kit by providing them with the skills and knowledge that helps them overcome their barriers. However, the qualitative findings do not explain the lack of association between patient experience and ordering of a kit at the end of a CFAK clinic.
How do interviews with CFAK attendees explain differences in patient experience by sociodemographic characteristics?	Patient experience did not differ by participant sociodemographics, apart from gender (F(1,64.25=9.55, p=0.003, ω^2^=0.097, 95% CI 0.004 to 0.228)	Theme 3: Relational and contextual factors that make CFAK effective Participants described how CFAK provided a positive, supportive healthcare experience through the HPOs’ approachable, knowledgeable, non-pressurising and informal approach. They saw the HPOs and clinics as responsive to their individual needs, offering personalised care and a safe space to ask questions. Information was made accessible through tailoring to the participant’s level of understanding and to the participants’ own language.	The quantitative findings suggest an equitable positive experience across participants from different sociodemographic backgrounds. The qualitative findings explain this by highlighting the relational and contextual factors that participants experienced during the CFAK clinics in which they felt supported, comfortable and information was tailored to them. The quantitative finding of a gender difference in patient experience is divergent to the qualitative findings as there was no indication of a different experience by gender in the interview analysis. This is likely due to the interview participants all having a positive experience. This is discussed as a limitation in the discussion section.

BCSP, Bowel Cancer Screening Programme; CFAK, Call for a Kit; CRC, colorectal cancer; FIT, faecal immunochemical test; HPOs, health promotion officers.

## Discussion

### Principal findings

This mixed-methods evaluation explored patient experiences of the CFAK intervention and identified mechanisms that support bowel screening uptake among non-responders. Quantitative findings showed that most participants had a positive experience. No differences were observed across most demographics or health characteristics, suggesting that CFAK is broadly acceptable and accessible. Notably, patient experience scores were significantly associated with intention to complete the screening kit, though not with kit ordering. Qualitative findings provided deeper insight into this distinction, revealing that CFAK addressed key barriers, such as low awareness, confusion about the kit and emotional discomfort, through personalised education, reassurance and culturally sensitive support. These relational and contextual factors helped build trust and confidence, particularly among those who were initially hesitant or anxious. Together, these findings suggest that while CFAK may not directly influence the decision to order a kit, it plays a role in enhancing psychological capability and motivation to follow through with screening. This highlights the importance of patient-centred communication and relational care in promoting health behaviours, especially in areas of low uptake in the UK.

### Comparison with existing literature

Our findings align with a growing body of evidence demonstrating that patient navigation and community-based interventions can improve cancer screening uptake, particularly among underserved groups.^26 27^ CFAK shares key features with these interventions, including personalised outreach, culturally sensitive communication and support in navigating screening processes. These elements have been shown to be especially effective in addressing barriers related to health literacy, mistrust and logistical challenges.^26 27^ However, this study adds to the literature in several important ways.

First, while previous evaluations of CFAK have demonstrated its effectiveness in increasing screening uptake,^7^ this is the first study to explore the mechanisms of impact from the patient perspective. Our findings suggest that CFAK enhances physical and psychological capability (eg, understanding the purpose and process of screening) and automatic motivation (eg, reducing fear and avoidance), and social opportunity (eg, through trusted, face-to-face interactions), all core components of the COM-B model.^16^ Second, we identified novel barriers not widely reported in previous studies, such as confusion between FIT kits and COVID-19 tests, and lingering distrust stemming from negative experiences with the older FOBt. Third, our behavioural analysis suggests that CFAK employs multiple behaviour change techniques, including instruction on how to perform the behaviour, reassurance, social support and tailoring, all of which are associated with improved screening behaviours.^26 28 29^ However, few studies have explicitly mapped these techniques in real-world interventions, and our findings contribute to this emerging area of implementation science. Finally, while many patient navigation programmes are delivered remotely or via telephone, participants in our study strongly valued face-to-face delivery, especially when conducted in their preferred language and tailored to their current level of understanding. This highlights the importance of relational care and cultural competence in designing equitable screening interventions within areas of low uptake.

### Strengths and limitations

A key strength of this study is its mixed-method approach, which allowed for triangulation of findings and a more nuanced understanding of patient experiences. The quantitative survey captured broad patterns in satisfaction and behavioural intent, while the qualitative interviews provided rich, contextualised accounts of how and why CFAK works. This integration strengthens the validity of the findings and informs future intervention design. The study is also subject to limitations. The survey response rate was low, and participants were self-selected as participants completing surveys opportunistically and independently as part of their care to support research and evaluation. Unfortunately, we identified that the survey distribution deviated from the planned protocol. The initial sample size estimate did not account for lower engagement within the target population, and operational challenges—including staff absence, reduced clinic attendance and inconsistent offering of surveys—limited recruitment. Despite discussions with CFAK team members, time constraints restricted the ability to fully address these issues. Consequently, only 500 surveys were distributed, which may limit the representativeness of the sample. This approach may reflect how a PEQ might be implemented in practice specific to community-centred interventions, proving a realistic reflection of the potential response rates, but limits generalisability. The low sample size also reduces statistical power, which may explain the lack of association between experience and kit ordering as well as with women reporting significantly higher satisfaction than men. Gender difference should be interpreted with caution as, while a medium effect size was found, scores on the PEQ were high for both men (M=31.23 out of 35) and women (M=33.40 out of 35). Further research is needed to understand if gender-concordant care as part of CFAK addresses women’s expectations from healthcare providers.^30^

Furthermore, social desirability bias could also be at play as it is possible that respondents ordered the test kit either because they had not formulated a decision at the CFAK consultation or because they did not want to be perceived as non-compliant. This could also explain why only half of CFAK attendees return their replacement test kit observed in the larger evaluation data.^7^ In regard to the representativeness of the qualitative sample in relation to the quantitative sample, age, gender and gender distribution were similar, but we only had two interviews with individuals from ethnic minority groups. While interviews in other languages (eg, Gujarati, Punjabi, Urdu) were offered and made possible, the limited response to the interviews from ethnically diverse survey respondents may be related to persistent sociocultural barriers, stigma and mistrust.^31^ This highlights the need to further consider how to make research inclusive and accessible for underrepresented communities, beyond offers of use of preferred language. Additionally, all but one interview participant had completed the kit following CFAK, limiting insight into the experiences of those who remain disengaged. These limitations, along with the exploratory nature of the study, mean that replication will be useful for further understanding of patients experience of CFAK clinics, with future evaluations seeking to recruit individuals who decline or do not complete screening after CFAK, to better understand persistent barriers.

### Implications for research and practice

Measuring patient experience is an important part of evaluating equitable service delivery. The CFAK PEQ has since been revised and implemented in practice to support ongoing monitoring and quality improvement. Findings have informed CFAK training, health equity assessments and audits, reinforcing the intervention’s effectiveness and inclusivity. With growing evidence and consensus on community-centred tailored interventions targeting inequalities in cancer screening programmes,^27 32^ much of the research is still focused on the effectiveness of interventions. While CFAK is effective in improving screening participation,^7^ it was important to embed a robust patient experience evaluation to understand the mechanisms that work, and what can be improved for implementing successful interventions in wider settings. Future studies should focus on validating the patient experience scale used to enable further evaluation of the implementation of CFAK in Lancashire and South Cumbria and its adaptation for inclusion groups (eg, people with learning disabilities) and other settings in England. This will enhance understanding around what factors are important for improving bowel screening uptake in non-responders across communities and areas of England. Furthermore, given the significant finding on patient experience and gender, further research should explore whether patient navigation is experienced/valued in different ways across genders.

### Conclusions

This mixed-methods evaluation provides new insights into how the CFAK intervention supports bowel cancer screening uptake among non-responders in underserved communities within England. While CFAK may not directly influence the decision to order a screening kit, it plays a critical role in enhancing psychological capability, motivation and confidence to complete it. The intervention’s relational and culturally sensitive approach helped address longstanding barriers, including low awareness, emotional discomfort and confusion about the screening process. By centring patient experience, this study highlights the value of tailored, inclusive and community-based strategies in reducing inequalities in cancer screening. Findings support the continued implementation and adaptation of CFAK across diverse settings and populations, particularly for areas with low uptake of CRC screening within England, and underscore the importance of embedding behavioural science and patient-centred design in future screening interventions.

## Supplementary material

10.1136/bmjopen-2025-109731 online supplemental file 1

## Data Availability

Data are available on reasonable request.
