# Supplementary material for: Understanding patient experiences of a community-based intervention to improve bowel screening uptake: a mixed-method evaluation of Call for a Kit clinics
Source: BMJ Open. 2026 Mar 24;16(3):e109731. doi: 10.1136/bmjopen-2025-109731 (PMC13034267; doi:10.1136/bmjopen-2025-109731)
Supplement: online supplemental file 1 [file bmjopen-16-3-s001.docx]

**Supplementary Materials**

**A mixed-method evaluation of a community-delivered intervention for improving bowel screening uptake**

**S1 Interview schedule**

**Interview questions for individuals who attend the Bowel Screening Clinic Version 1.1 11102023**

Hello, my name is Shahida and I work for the National Bowel Cancer Screening Programme. The purpose of this interview is to explore your experience at the bowel screening clinics at your GP practice, and how we can improve our service in the future. We would like to encourage you to go into as much detail as you can, what you have to say is very valuable information. Our interview/chat will last approximately 45 minutes. Everything you say is strictly confidential and anonymous. There are no right or wrong answers; I simply want to hear your views. I am not here to judge your comments but rather to collect them. Quotes may be used when the results of the study are being presented or published, but again you will not be personally identified.

Are you happy for me to record this interview? If No, will take notes (or terminate interview if notes are not allowed)

If Yes….. PRESS RECORD

Bowel Cancer Screening experience

I would like to start with talking about your previous experience of receiving a bowel cancer test kit in the post. You may have an idea of what we will be discussing from the information sheet I have sent you. We think that there are a lot of reasons affecting people’s decision to have screenings or not. I would like to encourage you to talk freely about everything that comes to your mind in relation to your experience with bowel cancer screening.

Would you be able to tell me about your thoughts on the kit and the bowel screening?

Prompts:

What did you think about the invitation materials? (Can you remember anything that caught your attention, can you remember any of the contents)

How did you make up your mind whether you wanted to take part?

How did completing/not completing the test make you feel?

Were there any practical issues?

Do you remember speaking to anyone about it?

(If they took part in previous screenings) Do you think there was a specific reason why on those/that occasion you did not take part in screening?

*Thank the participant so far for discussing their thoughts and feelings about screening freely.*

CFAK Phone call

As much as you can remember would you be able tell me about your thoughts and feelings when you first received a phone call to attend the bowel screening clinics?

Prompts:

Can you remember being called by a member of the Bowel Screening Team?

Do you remember your initial reaction when you got the phone call?

What do you remember about that conversation with the person that called you?

How did it make you feel at the time being called up?

Was there any particular information that you remember that helped you make the decision to attend the clinic? (How did you decide to attend the appt?)

Experience at the clinic

Can you tell me about your experience at the clinic? (Prompt for instance the information provided, people who delivered it, what did you like/ not like)

Prompts:

What did think about the bowel screening clinics?

Do you feel that the information provided was relevant and tailored for you?

Do you have any thoughts about the people who delivered it?

Did you feel included/welcomed?

Was there any information you felt could have been delivered differently?

What was good about the clinic?

Was there anything not so good about the clinic that can be improved?

Learning outcomes

What did you think you have taken away from attending the clinic?

- You mentioned concerns or lack of knowledge about X,Y,Z (see comment on exploring their reason for non -p participation), would you be able to tell me in what your views have changed or what you have learnt from the experience?

Outcome after attending the clinic

Can you tell us about what happened after the clinic?

Prompts:

For instance, have you received the kit that was ordered at the clinic and what?

Have you completed the Kit – if yes why have you completed it?

If no – why did you not complete the kit?

**S2 Factor Analysis output**

| Table I Correlation Matrix for Patient Experience Items | | | | | | | | |
| --- | --- | --- | --- | --- | --- | --- | --- | --- |
|  | | ExperienceInformed | ExperienceTime | ExperienceValues | ExperiencePersonal | ExperienceConcerns | PO_ExperienceSatisfaction | ExperienceQuestions |
| Correlation | ExperienceInformed | 1.000 | .762 | .318 | .598 | .788 | .798 | .839 |
|  | ExperienceTime | .762 | 1.000 | .480 | .638 | .646 | .833 | .844 |
|  | ExperienceValues | .318 | .480 | 1.000 | .652 | .556 | .453 | .455 |
|  | ExperiencePersonal | .598 | .638 | .652 | 1.000 | .727 | .674 | .692 |
|  | ExperienceConcerns | .788 | .646 | .556 | .727 | 1.000 | .737 | .764 |
|  | PO_ExperienceSatisfaction | .798 | .833 | .453 | .674 | .737 | 1.000 | .862 |
|  | ExperienceQuestions | .839 | .844 | .455 | .692 | .764 | .862 | 1.000 |

| Table II Principal Component Analysis Eigenvalues for Patient experience scale | | | | | | |
| --- | --- | --- | --- | --- | --- | --- |
| Component | Initial Eigenvalues | | | Extraction Sums of Squared Loadings | | |
|  | Total | % of Variance | Cumulative % | Total | % of Variance | Cumulative % |
| 1 | 5.087 | 72.674 | 72.674 | 5.087 | 72.674 | 72.674 |
| 2 | .856 | 12.233 | 84.907 |  |  |  |
| 3 | .386 | 5.512 | 90.419 |  |  |  |
| 4 | .269 | 3.843 | 94.262 |  |  |  |
| 5 | .161 | 2.303 | 96.565 |  |  |  |
| 6 | .124 | 1.769 | 98.334 |  |  |  |
| 7 | .117 | 1.666 | 100.000 |  |  |  |
| Extraction Method: Principal Component Analysis. | | | | | | |

Figure I Scree Plot for Patient Experience Items
