# Supplementary material for: Understanding patient experiences of a community-based intervention to improve bowel screening uptake: a mixed-method evaluation of Call for a Kit clinics
Source: BMJ Open. 2026 Mar 24;16(3):e109731. doi: 10.1136/bmjopen-2025-109731 (PMC13034267; doi:10.1136/bmjopen-2025-109731)
Supplement: Supplementary file 2 [file bmjopen-2025-109731.reviewer_comments.pdf]

PEER REVIEW HISTORY

BMJ Open publishes all reviews undertaken for accepted manuscripts. Reviewers are asked to complete a checklist review form and are provided with free text boxes to elaborate on their assessment. These free text comments are reproduced below.

ARTICLE DETAILS

Title (Provisional)

Understanding patient experiences of a community-based intervention to improve bowel screening uptake: A mixed-method evaluation of Call for a Kit clinics

Authors

Hanif, Shahida; Jefferson, Rebecca; Kerrison, Robert; Stoffel, Sandro T.; Rowley, Stephen; Morris, Lorraine; von Wagner, Christian; Hirst, Yasemin

---

VERSION 1 - REVIEW

**Reviewer**	**1**
**Name**	**Drott, Jenny**
**Affiliation**	**Linköping University**
**Date**	**21-Oct-2025**
**COI**	**None**

The study aims to examine whether patient experience of CFAK is associated with ordering and intention to complete a kit, and to explore how CFAK addresses barriers to participation in bowel cancer screening. The topic is important.

Interesting and well-written introduction and background to your study.

A reflection on the sample, and that people over 54 years or older were included and how this year was chosen as the limit. Does this need to be clarified in relation to current evidence?

The missed invitation to the screening, how was this ensured in more detail. Can it be clarified further.

Don't understand the division of study 1 and 2, when it's a mixed method study. Isn't it the constituent parts of the study with different methods that are mixed. Does this need to be revised to a quantitative part and a qualitative part. Consider to add references that strengthen your choice of method, and what focus you have on mixed method, is it a parallel convergent design?

Why chosen STROBE reporting guidelines and checklist, and not GRAMMS?

Consider clarifying the qualitative part and analysis, as well as adding more information about reflexivity and to cite literature on credibility.

Would be valuable if a synthesis of the whole was visible in the result based on the fact that the study is a mixed method study. Now it is more presented part by part, which more reflects a multi-method study.

**Reviewer**	**2**
**Name**	**Jones, Michael**
**Affiliation**	**Macquarie University, School of Psychological Sciences**
**Date**	**12-Dec-2025**
**COI**	**None**

Thank you for the opportunity to review this manuscript by Hanif and colleagues. CRC screening is an important public health tool and any effort to understand low uptake has the potential to yield strong benefits in the future, the paper therefore deals with an important topic. This is a generally well-designed and reported study that has much to commend it, and I applaud the authors for tackling this subject. There are however some deficiencies in the manuscript as it currently stands, which are detailed below.

1. The patient experience measure appears to have been created and used in this study without any independent, prior validation. Since this forms an important element of the study's findings I feel this is a major limitation since we do not know that it is actually measuring what the authors believe it is measuring and therefore is prone to misinterpretation. If the authors have any capacity to evaluate convergent/divergent validity, discriminant validity, test-retest reliability, ... that information should be added to the manuscript. If this information is not available that would seem to be a major limitation.

2. I do not have a sense of what magnitude of difference in the patient experience measure would be considered meaningful or might correspond to a small/medium/large effect size. I would urge the authors to consider reporting a standardised measure of effect size, such as Cohen's d. An example of where this might matter is the last para on page 11 where the gender difference reaches statistical significance but the difference in mean scores (effect size) seems quite small to me, hence I am not sure whether gender really matters or not.

3. The authors report p-values from hypothesis tests that assume normality of the outcome measures, was this evaluated. Please give some detail.

4. Looking at Table 3, I am surprised that the descriptive statistics for Gender and Long Term Condition seems to be almost identical but the p-values differ by an order of magnitude, please check they are correct. The Long Term Condition factor does have a slightly smaller sample size, perhaps that explains the difference.

5. Were respondents able to choose >1 of the Inequalities listed in Table 1? It would be useful to say whether or not.

6. If I understand correctly, 12 of 113 patients consented to be interviewed. While this number is common for qualitative studies, the response rate is low. Can the authors provide some indication of whether the 12 interviewees were representative of the overall sample?

7. I am concerned that the authors have relied almost exclusively on p-values to make conclusions in Study 1. Given the small sample size and consequent potential for low statistical power, I think effect size should be considered as important as p-value. Some comment on statistical power would also be useful.

8. The authors report on numerous hypothesis tests in Study 1 but do not appear to have made any allowance for multiple hypothesis testing and consequent inflation of type I error. They should either apply a false discovery rate procedure or declare this to be an exploratory study whose findings are in need of replication.

9. The authors intentionally sampled from general practices where uptake of FIT kits was low, and this makes perfect sense. I would however then be careful in the Discussion of their findings to highlight that they apply to regions of the UK with similarly low uptake rates. Currently the Discussion reads as though it applies to the entire UK, to me at least.

VERSION 1 - AUTHOR RESPONSE

Reviewer 1

The study aims to examine whether patient experience of CFAK is associated with ordering and intention to complete a kit, and to explore how CFAK addresses barriers to participation in bowel cancer screening. The topic is important. Interesting and well-written introduction and background to your study.	We thank Dr. Jenny Drott for their positive assessment of the topic and introduction and background to the study.
A reflection on the sample, and that people over 54 years or older were included and how this year was chosen as the limit. Does this need to be clarified in relation to current evidence?	Thank you. We have clarified in the participants section (page 6-7) why this age limit was chosen. At the time of the study, the screening age for Lancashire's Bowel Screening Programme was 54-74 years so only people aged 54-74 were eligible for screening and subsequently for CFAK, providing they had missed their previous screening episode. The official screening age reduction to 50 (i.e., inviting 52- and 50-year-olds) was completed from May 2025.
The missed invitation to the screening, how was this ensured in more detail. Can it be clarified further.	The missed screening episode is a prerequisite to be invited to a CFAK clinic. For clarity and consistency with the literature, we reworded as "not completed" in text as it is different from a

	missed appointment. Study participants were attendees of the CFAK clinic (and recruited at the end of their appointment by the HPO) therefore CRC screening non-responders. We have attempted to clarify this in the setting section (page 5), by explaining the process when someone does not respond to their screening invite.
Don't understand the division of study 1 and 2, when it's a mixed method study. Isn't it the constituent parts of the study with different methods that are mixed. Does this need to be revised to a quantitative part and a qualitative part. Consider to add references that strengthen your choice of method, and what focus you have on mixed method, is it a parallel convergent design?	Thank you for your comment and suggestion here. We agree that the mention of study 1 and study 2 takes away from the mixed methods, when they are in fact part of the same study. We have removed mention of study 1 and 2 and instead referred to them as quantitative survey and qualitative interviews.<br>We have used a convergent parallel design and have added brief justification and reference to support this at the end of the introduction (page 4).
Why chosen STROBE reporting guidelines and checklist, and not GRAMMS?	Thank you for highlighting this. We had intended to also include the COREQ for the qualitative element. We have now included the COREQ and GRAMMS checklist in the supplementary materials and mentioned these in the Methods Design section (page 5).
Consider clarifying the qualitative part and analysis, as well as adding more information about reflexivity and to cite literature on credibility.	We have added more detail to the qualitative aspects where we think it would be beneficial while attempting to be concise in consideration of word count. We have also added some detail around reflexivity and added references. (pages 7-12)
Would be valuable if a synthesis of the whole was visible in the result based on the fact that the study is a mixed method study. Now it is more presented part by part, which more reflects a multi-method study.	We appreciate and agree that it seemed to reflect a multi-method study in the result section. We had tried to provide some integration in the discussion section but can see that this was not entirely clear. To address this, we have included a joint analysis table of the quantitative and qualitative findings with an interpretation. (see table 4, page 21-23). We have also added a mixed method analysis section in the methods section to also add clarity (page 12)

Reviewer 2

Thank you for the opportunity to review this manuscript by Hanif and colleagues. CRC screening is an important public health tool and any effort to understand low uptake has the	We thank Prof. Michael Jones for their positive assessment on the study design and importance of the topic.

potential to yield strong benefits in the future, the paper therefore deals with an important topic. This is a generally well-designed and reported study that has much to commend it, and I applaud the authors for tackling this subject. There are however some deficiencies in the manuscript as it currently stands, which are detailed below.	
The patient experience measure appears to have been created and used in this study without any independent, prior validation. Since this forms an important element of the study's findings I feel this is a major limitation since we do not know that it is actually measuring what the authors believe it is measuring and therefore is prone to misinterpretation. If the authors have any capacity to evaluate convergent/divergent validity, discriminant validity, test-retest reliability, ... that information should be added to the manuscript. If this information is not available that would seem to be a major limitation.	To our knowledge, there are no previously validated patient experience questionnaires for measuring patient navigation provided to non-responders in cancer screening programmes. Thus, we explored whether the questionnaire for this study can be developed using the questions provided to the individuals who have an abnormal screening result and are invited to a colonoscopy as part of the English Bowel Cancer Screening programme (BCSP). At the time of the study, the PROMS for BCSP were being reevaluated and codeveloped part of a PhD studentship. We selected appropriate questions in discussion with the CFAK team and obtained feedback from patient representatives. We have added more detail in the Methods section on how this measure was developed and what it includes to provide more transparency for readers (page 8-9).<br>Due to the nature of this study being an exploratory study and the low response rate during the evaluation period, we did not evaluate psychometric properties of the survey. However, the survey has been implemented in 2025 and currently being rolled out. Once sufficient responses are received, we agree that other psychometric components should be evaluated and validated for its long-term use. We have now included the need to validate the measure in the discussion implications for research and practice section (page 27).
I do not have a sense of what magnitude of difference in the patient experience measure would be considered meaningful or might correspond to a small/medium/large effect size. I would urge the authors to consider reporting a standardised measure of effect size, such as Cohen's d. An example of where this might matter is the last para on page 11 where the gender difference reaches statistical significance but the difference in mean scores (effect size) seems quite small to me, hence I am not sure whether gender really matters or not.	Thank you for this suggestion. After reading the literature relating to appropriate measures of effect size for ANOVA, we have included Omega squared as this is viewed as the least biased effect size compared to Eta squared especially when the sample size is small. This has been clarified in the Quantitative Analysis section (page 10-11). The effect sizes have been added to Table 3 (page 16) and acknowledgement of the effect size in relation to the significant gender result has been made on page 15. The effect size for gender is of medium size,

	however, given the seemingly small difference in mean values of patient experience between men and women we have urged caution around interpretation of this different in the discussion (page 26). As the maximum score on the scale is 35, both scores seem to be high (i.e., positive experience). We have also added a suggestion in the discussion that further research should further explore whether patient navigation is experienced/valued in different ways across genders (page 26).
The authors report p-values from hypothesis tests that assume normality of the outcome measures, was this evaluated. Please give some detail.	Levene's tests were used to assess homogeneity of variance for the ANOVA's. The Levene's test was violated for four of the five ANOVA analyses. For these analyses Welch's Anova was used. The legend of Table 3 indicates which of the analyses are Welch's ANOVA. We have also clarified in the 'Quantitative analysis' section that Welch's ANOVA was used when the Levene's test for normality of variance was violated (page 10).
Looking at Table 3, I am surprised that the descriptive statistics for Gender and Long Term Condition seems to be almost identical but the p-values differ by an order of magnitude, please check they are correct. The Long Term Condition factor does have a slightly smaller sample size, perhaps that explains the difference.	We also noticed this and think that it can be explained by the difference in sample size. We have checked the p-values and they are correct.
Were respondents able to choose >1 of the Inequalities listed in Table 1? It would be useful to say whether or not.	Yes they were, thank you for pointing that out. We have now clarified this in Table 1 (page 13) and in the 'survey data' section (page 8-9).
If I understand correctly, 12 of 113 patients consented to be interviewed. While this number is common for qualitative studies, the response rate is low. Can the authors provide some indication of whether the 12 interviewees were representative of the overall sample?	We added further details about recruitment of the survey respondents and the interview participants to describe the response rates to the evaluation in further detail. While age, gender and gender distribution is similar to rest of the respondents, we only had 2 interviews with individuals from ethnic minority groups despite the offer of having the interview in their preferred language. This is now included as a limitation (page 26).
I am concerned that the authors have relied almost exclusively on p-values to make conclusions in Study 1. Given the small sample size and consequent potential for low statistical power, I think effect size should be considered as important as p-value. Some comment on statistical power would also be useful.	Thank you – this relates to a previous comment on the 3rd row of this response table, which we have addressed there by adding in effect sizes. In regards to statistical power, we have added comment to this in the methods section (page 7) and in the discussion limitations section (page 26).
The authors report on numerous hypothesis tests in Study 1 but do not appear to have	Thank you for indicating that the exploratory nature of the study was not clear. We have tried

made any allowance for multiple hypothesis testing and consequent inflation of type I error. They should either apply a false discovery rate procedure or declare this to be an exploratory study whose findings are in need of replication.	to make this clearer at relevant points throughout the manuscript and have stated the need for replication in the discussion section (page 27).
The authors intentionally sampled from general practices where uptake of FIT kits was low, and this makes perfect sense. I would however then be careful in the Discussion of their findings to highlight that they apply to regions of the UK with similarly low uptake rates. Currently the Discussion reads as though it applies to the entire UK, to me at least.	Thank you, this is a good point. We have added clarification throughout the 'Discussion' section to address this.
